# Derrick Sim: funding pandemic response

**DOI:** 10.2471/BLT.24.030524

**Published:** 2024-05-01

## Abstract

Derrick Sim talks to Gary Humphreys about the funding lessons learned in the establishment and running of COVAX, and what is required to prepare for the next pandemic.

*Q: You started out as a doctor. How did you come to focus on the funding side of the vaccine access issue?*

A: I spent close to seven years working in paediatrics, and was also involved as a researcher on some Phase III clinical trials for vaccines early on in my career. I subsequently worked in various scientific and medical roles within the vaccines industry, contributing to the development of flu vaccines, among other products, including the H1N1 (influenza A virus subtype H1N1) pandemic flu vaccine. As far as the funding/financing aspect is concerned, I did an MBA after my medical degree and, before joining Gavi, spent nearly two decades primarily focused on vaccine commercialization.

*Q: To what extent did those experiences inform your work on ensuring access to COVID-19 vaccines?*

A: Significantly. For instance, I was in Asia during the SARS (severe acute respiratory syndrome) coronavirus outbreak in 2003, where, in the absence of a vaccine, containment efforts relied heavily on non-medical interventions such as testing, treating and isolating people with symptoms. Fortunately, that worked in most cases since most of the transmission was during the symptomatic phase. I also worked through the H1N1 influenza pandemic, when a vaccine was developed, but many low- and middle-income countries were unable to secure sufficient doses. These experiences really sharpened my focus on the question of vaccine access at Gavi, where my work has included leading efforts to engage with manufacturers to secure vaccines for the COVAX facility.

*Q: COVAX was wound up in December 2023. Can you describe in simple terms what it was and what it did?*

A: COVAX was the vaccines pillar of the Access to COVID-19 Tools Accelerator, a global initiative co-led by CEPI (the Coalition for Epidemic Preparedness Innovations), Gavi, WHO (the World Health Organization) and UNICEF (the United Nations Children’s Fund). Officially launched in April 2020, COVAX’s main goal was to ensure that all countries, regardless of income level, could access COVID-19 vaccines. Participating countries pooled their resources to support the development, manufacture and equitable distribution of COVID-19 vaccines. COVAX also facilitated negotiations with vaccine manufacturers to secure doses at affordable prices for all participating countries.

*Q: COVAX has been criticized for failing to meet its own targets and broader equity goals. How do you respond to that criticism?*

A: Despite facing systemic barriers such as vaccine nationalism and hoarding, by the end of last year COVAX had delivered nearly 2 billion doses of vaccines to 146 economies (countries, states and territories), achieving two-dose coverage of 57% in lower-income economies, where it is estimated to have averted some 2.7 million deaths. The majority of the vaccines were delivered to economies that needed them the most, with 74% being delivered to low-income economies. It’s also important to note that although COVAX was wound up, low- and lower-middle-income economies will continue to receive COVID-19 vaccines and delivery support from Gavi in 2024 and 2025, with 83 million doses so far requested for 2024 from 58 economies.

*Q: What for you have been the main lessons learned from the COVAX initiative regarding funding?*

A: Well to begin with, it would be hard to overstate the importance of having contingency funding in place to finance a rapid response. In an ideal world, outbreak response would happen the moment an outbreak occurred within health systems that are monitoring the emergence of novel pathogens in close-to real time. In the absence of such systems in many parts of the world, outbreak response usually takes the form of an abrupt capacity *surge*, generally supported by global health agencies in collaboration with regional and country partners. For that surge to happen, contingency funding needs to be in place and available from day one. It’s also important to note that front-loaded contingency funding doesn’t just permit a rapid response, it also gets around the need for multiple rounds of fundraising once a crisis is unfolding in what can be stressful circumstances. This ensures rapid implementation of programme operations and enhances adaptability.

*Q: What kind of contingency funding was in place at the beginning of the pandemic?*

A: There was no contingency funding in place at the outset of the pandemic. Gavi had to take several risks and proactive steps that included reallocating funds from core budgets to support immediate response efforts. Gavi also financed the set-up and staffing of COVAX before any dedicated funding was raised, and underwrote financial and legal agreements with Facility participants. As for COVAX partners – who, by the way, also had to tap into core budgets – they provided at-risk “push” funding (incentives supporting research and development) to vaccine innovators in the form of forgivable loans, and at-risk “pull” funding (incentives supporting product market entry) in the form of AMCs (advance market commitments).

*Q: Can you explain what AMCs are in simple terms?*

A: Essentially, AMCs guarantee a market for vaccines by committing funds for vaccine purchase in advance of vaccine licensure, thereby reducing the financial risks for vaccine manufacturers. Gavi has accumulated considerable expertise in developing such mechanisms through initiatives such as the pneumococcal conjugate vaccine AMC, which was launched by Gavi in 2009 with the support of the donor governments, vaccine manufacturers, and other stakeholders, to accelerate the availability of pneumococcal conjugate vaccines for developing countries, particularly for children in low-income settings at high risk of pneumococcal diseases. The Gavi COVAX AMC was launched in June 2020, and played a vital part in enabling us to supply vaccines free of charge to 87 low- and lower-middle-income economies. These early investments and actions to accelerate access meant that a number of institutions had to take on considerable risks in the face of significant unknowns relating to R&D outcomes and the trajectory the pandemic was going to take.

*Q: How ready were they to take such risks onto their balance sheets?*

A: There were some interesting conversations. Not everyone had the same risk appetite or capacity – sovereign states generally being more able to take on risk, and public health agencies and organizations less so. So, this was another key learning, and going forward, we need to establish clear risk thresholds through pre-negotiated agreements among partners, donors, and stakeholders to be able to make these commitments as quickly as possible.

*Q: Will Gavi also be adapting based on the lessons learned?*

A: Certainly, and we already have in some respects. For example, in 2022 we set up the Pandemic Vaccine Pool (PVP), the main aim of which was to secure additional contingency funds ahead of anticipated needs. The pool has supported a range of access initiatives, and is currently empowering countries seeking to sustain vaccine protection for vulnerable populations through 2024 and 2025. We've also leveraged partnerships with entities such as the European Investment Bank (EIB) and the United States Development Finance Corporation (DFC) to establish financial mechanisms designed to accelerate the flow of funding. During discussions at Gavi’s Global Vaccine Impact Conference that took place in June 2023, the EIB announced an extension of its support which entailed front-loading grants to Gavi aimed at bolstering routine and outbreak vaccination programmes. This included a 1 billion euros (1.08 United States dollars (US$)) financing facility designated for contingency grants. Similarly, the DFC is exploring avenues to support various immunization initiatives, including outbreak vaccines.

*Q: It sound like significant amounts are being committed. How close do they come to meeting anticipated need?*

A: Predicting the exact size or requirements for future emergencies is obviously challenging, as is the likely cost of responding to them. But one thing seems clear: not committing adequate funds will entail greater costs. It has been estimated that, worldwide, COVID-19 cost the global economy around US$ 29 trillion. Set against that kind of number, the billions committed to the different initiatives mentioned or, for example, to the World Bank's Pandemic Fund, which is targeting US$ 2 billion, seem relatively small.

*Q: Are we better prepared now than we were in 2020?*

A: I think so. Drawing on the foundation of knowledge and capabilities accumulated over 20 years of operation in routine immunization environments, including initiatives like the pneumococcal conjugate vaccine AMC, Gavi and its partners have been able to adapt, notably by leveraging their expertise in innovative financing and market shaping. We have also seen a significant improvement in health system capacity. This is partly as a result of the US$ 1.3 billion in funding that we have provided to 88 lower-income countries under the COVID-19 delivery support funding initiative. For example, we have seen a big improvement in cold chain infrastructure, including investments in cold rooms, refrigerators and refrigerated trucks. This investment is expected to have a lasting impact on routine vaccination efforts, as is the increased digitization of immunization registries and records. There has also been progress in the diversification of manufacturing networks, notably impacting African and regional vaccine manufacturing.

*Q: The pandemic response has sometimes been criticized as having been too focused on transmission prevention, at colossal socioeconomic and public health cost. What can be done to ensure that all points of view regarding response prioritization are heard in the next pandemic?*

A: That’s a tough question. We've come to realize the delicate balance required in decision-making during emergencies. On one hand, the need for agility and the ability to make swift decisions despite incomplete information or uncertain outcomes is paramount. However, we also recognize the importance of inclusiveness in decision-making processes, particularly involving participating countries and civil society. During the pandemic, Gavi engaged in regular discussions with civil society organizations to ensure transparency and keep stakeholders informed. While such initiatives were helpful, it’s clear that there is a need for continual reflection and improvement in these processes if we are going to do better in future emergencies.

